# Empowering tomorrow's leaders: the impact of the 15th Network of Young Researchers in Andrology (NYRA) meeting on male reproductive health and interdisciplinary collaboration

**DOI:** 10.1242/bio.060178

**Published:** 2024-01-05

**Authors:** Daniel Marcu, Dorte L. Egeberg, Guillaume Richer, Brendan Houston, Emily Delgouffe, Gülizar Saritas, Omar Ammar, Lydia Wehrli, Cyril Djari, Alberto de la Iglesia

**Affiliations:** ^1^ Network for Young Researchers in Andrology (NYRA), 08021 Barcelona, Spain; ^2^ School of Biological Science, University of East Anglia, NR4 7TJ Norwich, UK; ^3^ European Sperm Bank Struenseegade 9, 2, 2200 Copenhagen, Denmark; ^4^ Biology of the Testis lab, University Medical Campus, Vrije Universiteit Brussel, 1090 Brussels, Belgium; ^5^ School of BioSciences, The University of Melbourne, Parkville, Victoria, 3010, Australia; ^6^ University Department of Growth and Reproduction, Section GR-5064, Copenhagen University Hospital, Rigshospitalet, Blegdamsvej 9, DK-2100 Copenhagen, Denmark; ^7^ Ar-Razzi Hospital, 8968+JF2 Baghdad, Iraq; ^8^ Department of Genetic Medicine and Development, Faculty of Medicine, University of Geneva, CH-1211 Genève 4, Switzerland; ^9^ Université Paris Cité, INSERM, CNRS, Institut Cochin, F-75014 Paris, France

**Keywords:** European academy of andrology, Network for young researchers in andrology, *In vitro* spermatogenesis, Male infertility, Mammalian reproduction, Sperm physiology

## Abstract

The 15th Network of Young Researchers in Andrology (NYRA) meeting, held at the Palace de Caux, Switzerland, served as a valuable platform to disseminate cutting-edge research and facilitate interactions among early-career researchers and trainees in andrology from around the world. Preceding the 22nd European Testis Workshop, the 2-day event brought together participants from a variety of countries to discuss a range of topics pertaining to men's reproductive health and biology. Specific focuses included piRNAs in mammalian reproduction, biomolecules enhancing sperm physiology, advances in *in vitro* spermatogenesis, reproductive strategies across species, and career development. A dedicated ‘scientific speed-dating’ social event also stood out, encouraging cross-disciplinary collaborations and strengthening ties within the scientific community. The high participation rate of the meeting highlighted its value in connecting the andrology community. Finally, the announcement of NYRA's merger with the European Academy of Andrology (EAA) marked a pivotal moment, enabling NYRA to support young researchers while collaborating with the EAA to advance andrology research. The 15th NYRA meeting played a crucial role in enhancing knowledge dissemination and andrology research, empowering young researchers, and addressing key challenges in male infertility.

## Teaser

**Summary:** This paper provides an overview of the 15th NYRA meeting. The paper provides a general background and justification for the existence of our network.

## Introduction

The prevalence of male infertility, affecting approximately 9–16% of men, has prompted a need for improved diagnostic methods and interventions (Barratt et al., 2017). The conventional method of its diagnosis involves examining semen parameters (WHO, 2023), where sperm count has decreased by over 50% in the last 40 years, with a continuous decline of 2.6% per year among Western men (Barratt et al., 2017; Levine et al., 2023). This decline not only impacts fertilization rates but also leads to increased disease burden and healthcare costs (Hubens et al., 2018; Kasman et al., 2020; Levine et al., 2017). The root causes of male infertility are complex and diverse, including (epi)genetics, psychology, lifestyle, pathogens, and environmental insults (such as xenobiotics, oxidative stress, heat stress and chemotherapeutic agents) – all of which require further research to better understand their contributions (Aitken, 2022). However, for a significant number of male patients, the exact etiology remains unknown, as it can be assigned to only 30–50% of cases. Indeed, the poor understanding of male reproductive health hinders progress in research, diagnosis, and treatment of male infertility (Barratt et al., 2018; Duffy et al., 2021; Gumerova et al., 2023).

The integration of cutting-edge omics technologies, alongside the continuous emergence of novel analytical and visualization tools coupled with machine learning, promises to revolutionize our understanding of male infertility. Together, these approaches will aid in elucidating key infertility biomarkers as well as the underlying intricacies of cellular heterogeneity within the male reproductive system at an unprecedented level, providing a faster and more comprehensive understanding. Promising candidates are emerging as novel biomarkers associated with male infertility as well as with recurrent pregnancy loss, often overlooked by traditional analyses. Together with novel cell culture methodologies (Richer et al., 2020), testicular organoids will allow for mechanistic studies to address and understand (sub)cellular and (epi)genetic biomarkers on testis development and functions (i.e. androgenesis and spermatogenesis) (Alves-Lopes et al., 2017). These techniques can be used for scaled and controlled experimental settings, possibly leading to effective solutions that prevent/treat disease and/or improve the male reproductive diagnosis and care.

The widespread societal impact and the slow medical and regulatory advancements underlines the importance of prioritizing the challenge of male infertility. Therefore, initiatives like the Network for Young Researchers in Andrology (NYRA) have been established. NYRA aims to play a pivotal role in engaging younger stakeholders, raising awareness, and advancing research in andrology and reproductive biology. Collaborative efforts within research consortia are crucial for harnessing the full potential of emerging technologies in advancing male reproductive research.

## Organization of the 15th NYRA Meeting

**Fig. 1.**
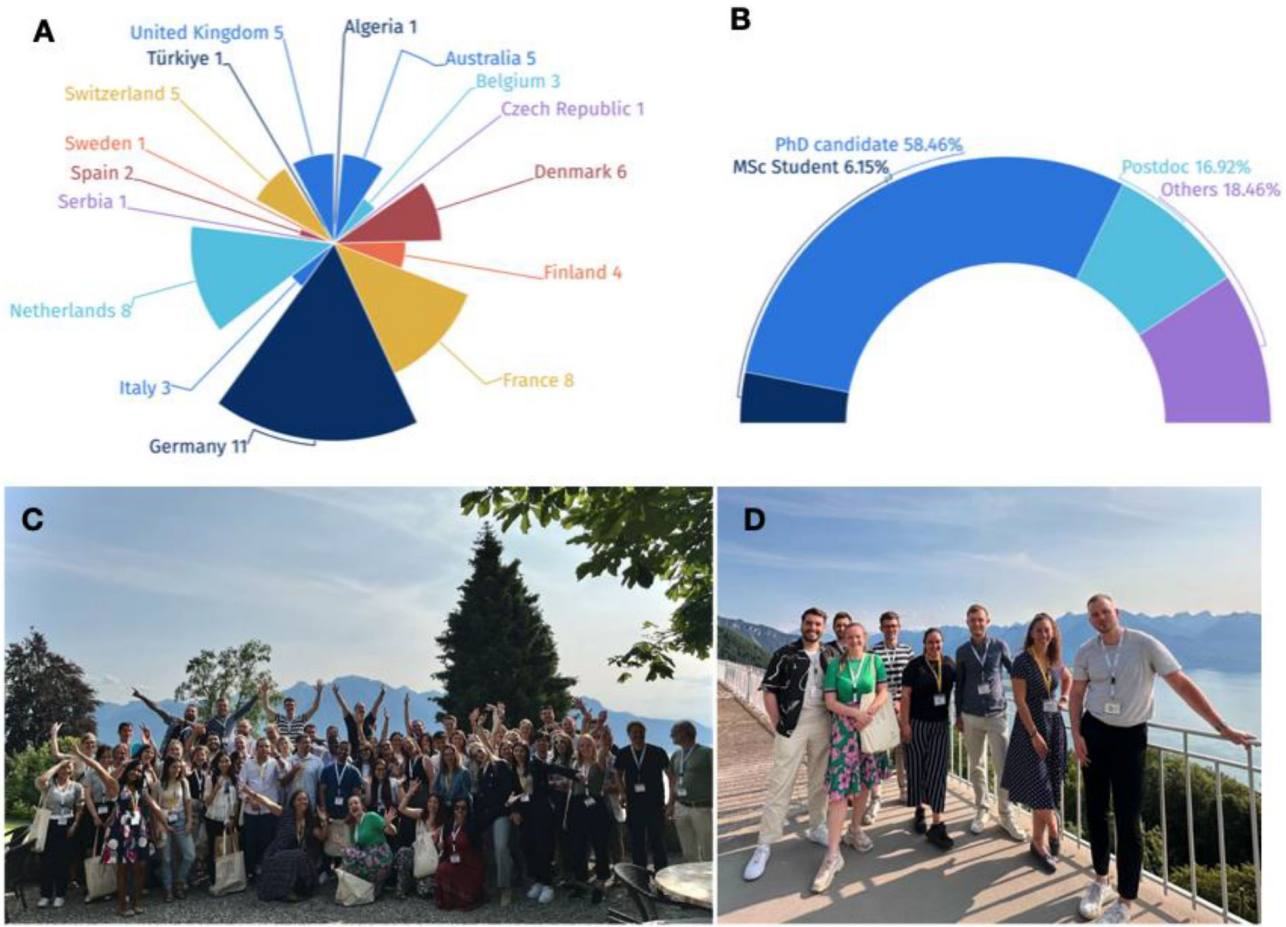
**Participants of the 15th NYRA meeting.** 15th NYRA delegates distributed by (A) country and (B) career stage. (C) Early-career scientists and trainees in andrology from around the world. (D) NYRA board members and local organizers.

The 15th NYRA meeting was held over two days (June 17–18, 2023) at the Palace de Caux, Switzerland, as a satellite event preceding the 22nd European Testis Workshop (ETW) that was co-organized by COST Action Andronet (CA20119) (Oliva et al., 2022). In total, 65 attendees representing 15 different countries (Algeria, Australia, Belgium, Croatia, Czech Republic, Denmark, Finland, France, Germany, the Netherlands, Poland, Spain, Serbia, Türkiye, and the UK) joined the meeting, mostly comprised of early-career researchers and trainees (Fig. 1A–C). The scientific program featured five prominent plenary speakers who covered diverse topics in andrology ranging from molecular aspects of male fertility to *in vitro* spermatogenesis and sperm physiology. In addition, the board organized a career development session focused on entrepreneurship. By disseminating cutting-edge research and facilitating interactions among young researchers, the 15th NYRA meeting facilitated knowledge exchange and networking opportunities, while strengthening ties within the scientific community. This was exemplified by the scientific speed-dating event, where participants, including the invited senior speakers and members of the NYRA board, engaged in dynamic discussions about their research and careers. Finally, the merger between NYRA and the EAA was announced, marking a significant milestone for the andrology field enhancing opportunities for young trainees through collaborative efforts.

At the time of the 15th NYRA Meeting, the NYRA Board was formed by Dr Alberto de la Iglesia (President), Dr Dorte Egerberg (Secretary), Daniel Marcu (Acting Treasurer), Guillaume Richer, Dr Brendan Houston, Emily Delgouffe, Gülizar Saritas, Omar Ammar and the local organizers Lydia Wehrli and Dr Cyril Djari (Fig. 1D).

## Scientific highlights of the 15th NYRA Meeting

**Fig. 2.**
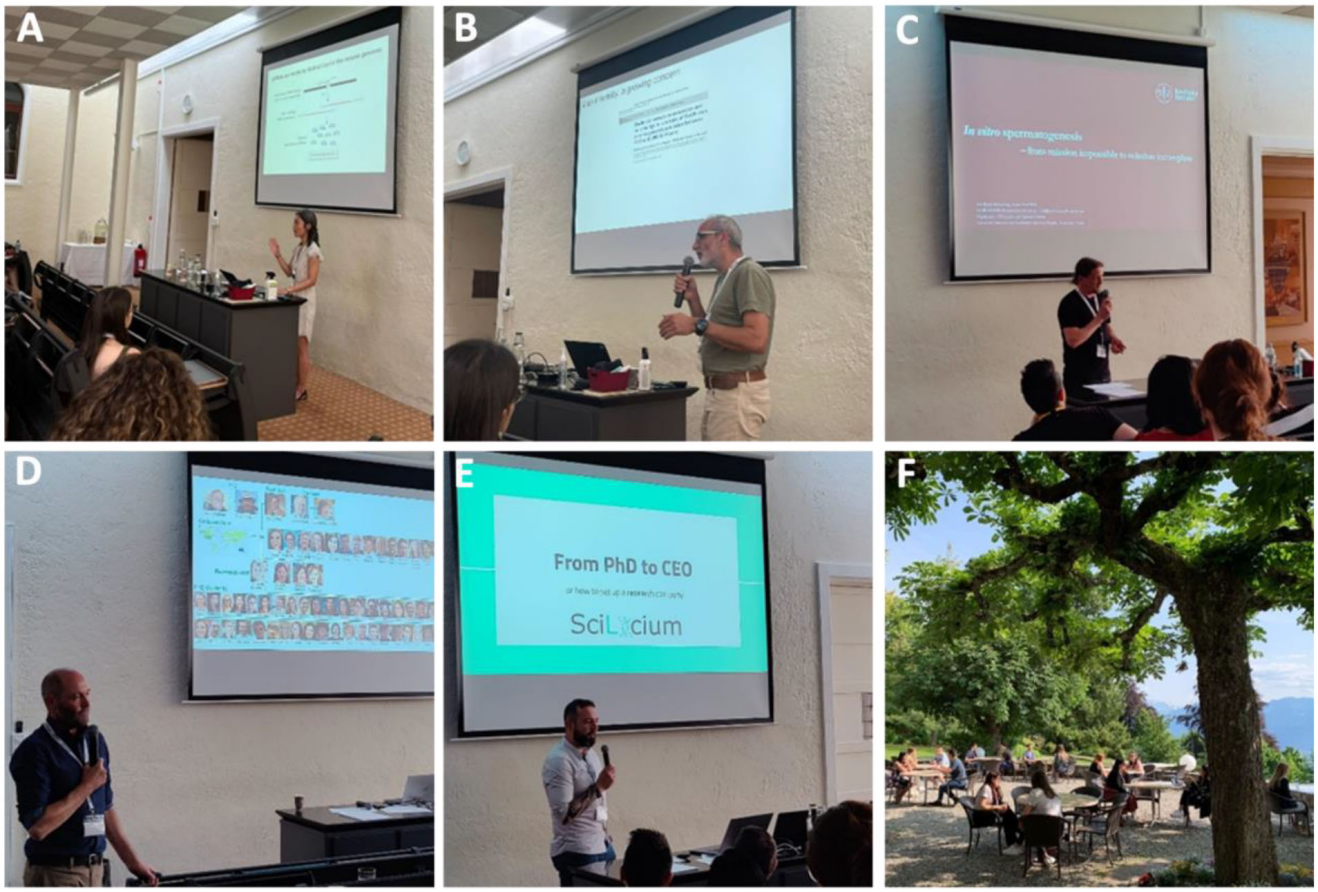
**Sessions of the 15th NYRA meeting.** (A–D) Plenary sessions. (E) career development workshop. (F) Speed-dating event.

Associate Professor Pei-Hsuan (Xuan) Wu (Genetic Medicine and Development, University of Geneva, Switzerland) delivered the inaugural plenary lecture entitled ‘PIWI-interacting RNAs in mammalian reproduction’ (Fig. 2A). Professor Pei-Hsuan provided us with an insight into a type of non-coding RNAs known as PIWI-interacting (pi) RNAs and their corresponding precursors in mice. The role of specific piRNA clusters was discussed though elimination of mouse piRNAs using CRISPR/Cas technologies. The functions of the pi6 cluster, particularly beyond gamete production, were revealed to extend into post-testicular sperm maturation.

Professor Christophe Arnoult (Institute for Advanced Biosciences, University of Grenoble Alpes, France) delivered a lecture entitled ‘In search of (bio)molecules that improve sperm physiology’ (Fig. 2B). Cryopreservation may have adverse effects on sperm competence, potentially triggering capacitation, provoking acrosome reactions, diminishing sperm motility, and thereby reducing the numbers of fertilization-competent spermatozoa. Considering the limited efficacy observed in assisted reproductive technologies (ART) and the cryopreservation-induced impacts on sperm physiology, Professor Arnoult presented a series of promising results centered on elucidating sperm functionality. These studies identified specific sperm proteins of interest as prospective targets for biomolecular interventions. Furthermore, he delved into the potential efficacy of directing therapeutic strategies toward these sperm-specific proteins to enhance the fertilization rates within ART.

Professor Jan-Bernd Stukenborg (NORDFERTIL Research Lab Stockholm, Childhood Cancer Research Unit, Department of Women's and Children's Health, Karolinska Institutet, Sweden) shifted the focus from sperm physiology to the production of testicular organoids, mini-testes in Petri dishes (Fig. 2C). During his lecture, titled ‘*In vitro* spermatogenesis – from mission impossible to mission incomplete’, he discussed the advancements made in the development of testicular organoids, which aims to create a physiological milieu capable of supporting *in vitro* spermatogenesis. Notably, the important role of Sertoli cells in supporting this complex process was highlighted.

The final plenary lecture delivered by Professor Brett Nixon (Priority Research Centre for Reproductive Science, School of Environmental and Life Sciences, University of Newcastle, Australia) with the title ‘What makes the best swimmers?’ (Fig. 2D), provided insights into reproductive strategies and fertility across various species, with a focus on epididymal sperm maturation. Reptiles, birds and monotremes such as the platypus displayed distinct differences in sperm structure and maturation compared to eutherians and humans. For example, reptiles such as female snakes are able to store functional sperm for up to 5 years before sperm were used for fertilization that resulted in pregnancy. Moreover, the lecture showcased adaptations of platypus sperm, which form bundles inside the female reproductive tract system to bolster their motility and aid in ascension of the oviduct.

## Career development session

As part of the traditional activities organized by NYRA, the board arranged a workshop focused on showcasing the process of launching a research-oriented startup company. For this, we welcomed Dr Thomas Darde, former NYRA board member and the current founder of SciLicium, based in Rouen, France (Fig. 2E). SciLicium focuses on redefining the field of bioinformatics by connecting individuals skilled in data analysis with those possessing expertise in data interpretation. They offer a personalized approach to assist their customers in optimizing their abilities in analyzing multi-omics data, providing computational workflows and tools required to aid address the challenges of modern scientific research. Additionally, SciLicium actively supports and encourages its clients to develop strong working relationships and partnerships with experts in their respective fields of study. Dr Darde provided valuable insights into the intricacies of initiating a company following the completion of doctoral studies. During his presentation, he presented his personal journey shifting from academia to founding SciLicium. He delved into the challenges that come with initiating a company, highlighting the diverse roles he had to fulfill along the way – including those of a bioinformatician, accountant, sales and marketing manager, among others. To excel in such a multifaceted role, he emphasized the importance of being present and adaptable, with a constant focus on error reduction. Moreover, Dr Darde elaborated on the complexity of being a leader and its responsibilities within a team of collaborators, underscoring the utilization of skill sets acquired throughout his academic career and during the pursuit of his PhD studies. Finally, he stressed the necessity of dataset sharing through collaborative efforts. The ReproGenomics Viewer exemplifies this collaborative approach as a multi- and cross-species genomic database designed for the visualization, mining, and comparison of published omics data sets, specifically tailored to serve the reproductive science community.

## Scientific speed-dating

One of NYRA's popular and highly anticipated networking activities is the ‘scientific speed-dating’ event (Fig. 2F). It was an incredibly rewarding experience that allowed NYRA members to connect on a meaningful level. The event was organized in a dynamic format: attendees were asked to randomly disperse across tables in the courtyard, and they had five minutes to provide a brief overview of their research and current career stage to a fellow participant before moving on to meet someone new. In total, each individual had the opportunity to engage with 10 different people during this event, breaking down barriers and fostering potential collaborations and discussions.

## NYRA history and announcement of the merger with the EAA

**Fig. 3.**
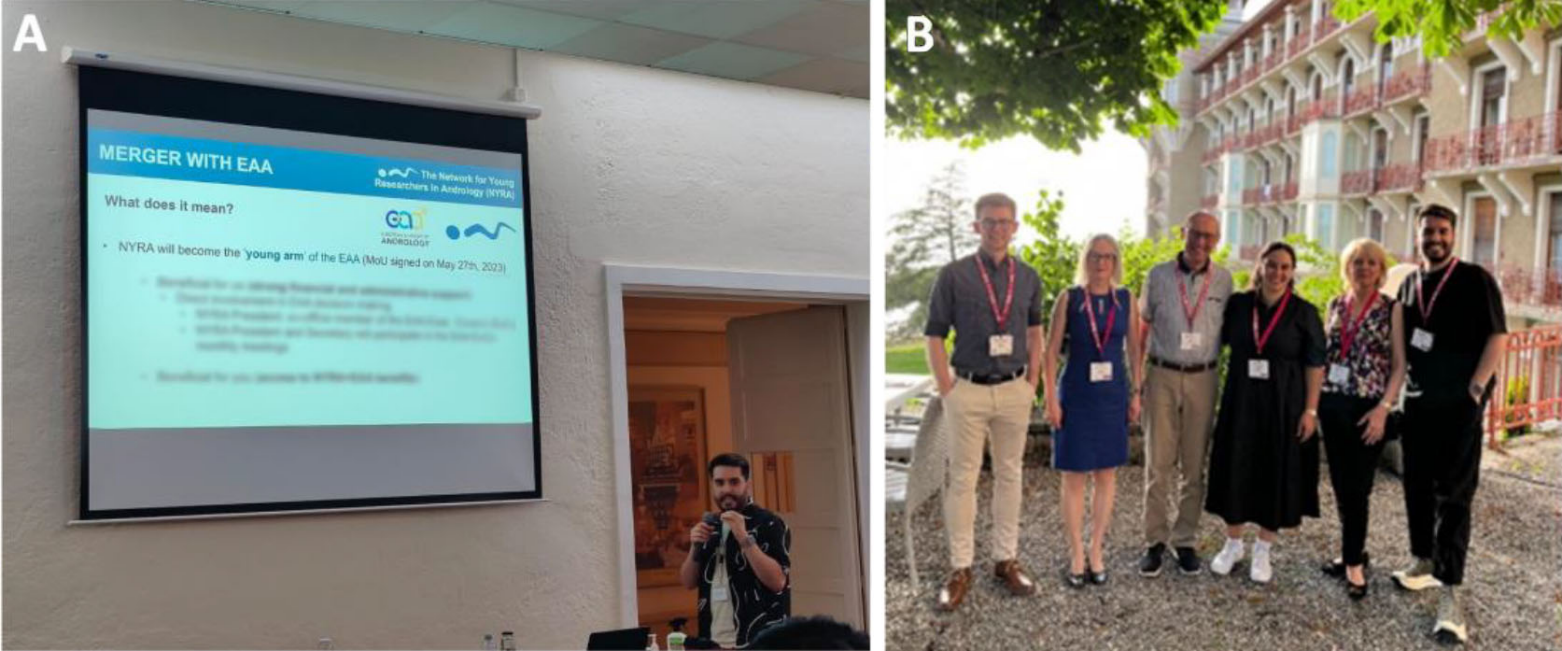
**NYRA–EAA merger.** (A) Merger announcement by NYRA president. (B) NYRA and EAA board members.

Dr Alberto de la Iglesia, NYRA president, delivered an insightful presentation that traced the history of NYRA, from its foundation in 2006 during the 14th ETW up to the present day (Fig. 3A), highlighting the valuable contributions and achievements of previous NYRA board members. Notably, he announced NYRA's latest milestone, the merger between NYRA and the European Academy of Andrology (EAA), with NYRA becoming the ‘young arm of the EAA’ while preserving its independent identity (Fig. 3B). The opportunity to share this merger announcement with the audience was a moment of immense satisfaction for all NYRA board members. As highlighted by Dr de la Iglesia, now also *ex officio* member of the EAA Executive Council, this development is a significant breakthrough for the andrology field, aiming to enhance opportunities for early-career researchers, including trainees, through collaborative efforts.
